# Fulvestrant-induced expression of ErbB3 and ErbB4 receptors sensitizes oestrogen receptor-positive breast cancer cells to heregulin β1

**DOI:** 10.1186/bcr2848

**Published:** 2011-03-11

**Authors:** Iain R Hutcheson, Lindy Goddard, Denise Barrow, Richard A McClelland, Hayley E Francies, Janice M Knowlden, Robert I Nicholson, Julia MW Gee

**Affiliations:** 1 Department of Pharmacology, Radiology & Oncology, School of Medicine, Cardiff University, Heath Park, Cardiff, CF14 4XN, UK; 2 Tenovus Centre for Cancer Research, Welsh School of Pharmacy, Cardiff University, King Edward VII Avenue, Cardiff, CF10 3NB, UK

## Abstract

**Introduction:**

We have previously reported that induction of epidermal growth factor receptor and ErbB2 in response to antihormonal agents may provide an early mechanism to allow breast cancer cells to evade the growth-inhibitory action of such therapies and ultimately drive resistant cell growth. More recently, the other two members of the ErbB receptor family, ErbB3 and ErbB4, have been implicated in antihormone resistance in breast cancer. In the present study, we have investigated whether induction of ErbB3 and/or ErbB4 may provide an alternative resistance mechanism to antihormonal action in a panel of four oestrogen receptor (ER)-positive breast cancer cell lines.

**Methods:**

MCF-7, T47D, BT474 and MDAMB361 cell lines were exposed to fulvestrant (100 nM) for seven days, and effects on ErbB3/4 expression and signalling, as well as on cell growth, were assessed. Effects of heregulin β1 (HRGβ1) were also examined in the absence and presence of fulvestrant to determine the impact of ER blockade on the capacity of this ErbB3/4 ligand to promote signalling and cell proliferation.

**Results:**

Fulvestrant potently reduced ER expression and transcriptional activity and significantly inhibited growth in MCF-7, T47D, BT474 and MDAMB361 cells. However, alongside this inhibitory activity, fulvestrant also consistently induced protein expression and activity of ErbB3 in MCF-7 and T47D cells and ErbB4 in BT474 and MDAMB361 cell lines. Consequently, fulvestrant treatment sensitised all cell lines to the actions of the ErbB3/4 ligand HRGβ1 with enhanced ErbB3/4-driven signalling activity, reexpression of cyclin D1 and significant increases in cell proliferation being observed when compared to untreated cells. Indeed, in T47D and MDAMB361 HRGβ1 was converted from a ligand having negligible or suppressive growth activity into one that potently promoted cell proliferation. Consequently, fulvestrant-mediated growth inhibition was completely overridden by HRGβ1 in all four cell lines.

**Conclusions:**

These findings suggest that although antihormones such as fulvestrant may have potent acute growth-inhibitory activity in ER-positive breast cancer cells, their ability to induce and sensitise cells to growth factors may serve to reduce and ultimately limit their inhibitory activity.

## Introduction

The ability of antihormones to inhibit growth of oestrogen receptor (ER)-positive breast cancer cells has principally been attributed to the ability of these agents to block the transcriptional activity of the ER and prevent activation of genes responsible for mediating cell cycle progression, such as cyclin D1 and c-Myc [1]. However, more recent findings have demonstrated that the majority of oestrogen (E2)-regulated genes in human breast cancer cells are repressed rather than activated and that many of these genes act as growth and/or transcriptional repressors [2]. Thus, antihormones may exert their antiproliferative activity not only through suppression of growth-promoting genes but also through an ability to induce negative regulators of cell proliferation. Although such an inductive mechanism will undoubtedly enhance the growth inhibitory activity of antihormones in ER-positive breast cancer, there is now evidence that this inductive capacity may be a double-edged sword, as it has also been demonstrated that E2 can suppress, and antihormones can induce, genes that promote cell proliferation and survival [3-5].

Two key proproliferative survival genes that have been established as E2-suppressed antihormone-induced genes in a range of ER-positive breast cancer cell lines are the ErbB receptors epidermal growth factor receptor (EGFR) and ErbB2 [2-4,6-11]. As there is considerable preclinical and clinical evidence that both EGFR and ErbB2 play a central role in driving acquisition of antihormone resistance in breast cancer [12-19], it is possible that antihormones may actually play an active role in limiting their own activity through an ability to promote expression of these potent growth promoters. Indeed, there is now evidence based on *in vitro *and *in vivo *MCF-7 cell models of ER-positive breast cancer that increased expression of EGFR and ErbB2 is an early response to tamoxifen treatment and that this induction of ErbB signalling maintains residual activity of key downstream signalling pathways, such as the mitogen-activated protein kinase (MAPK) and phosphatidylinositol 3-kinase (PI3K) cascades [10,13]. Such signalling may allow cells to evade inhibition during the drug-responsive phase, as targeting these ErbB receptors in combination with tamoxifen suppresses the residual signalling activity and greatly improves and extends the growth-inhibitory action of this antihormone in the two cell models [10,13]. Similar preclinical findings have also been reported for the pure anti-ER fulvestrant [10], and recent clinical studies examining cotargeting ER and EGFR and/or ErbB2 signalling pathways have reported improved response to a range of endocrine therapies in breast cancer patients [20,21]. Despite these positive findings in clinical trials, it should also be noted that a considerable number of patients do not benefit from such combination treatments, suggesting that alternative EGFR- and ErbB2-independent mechanisms of resistance to antihormones are active in the clinical setting [20].

Microarray studies have now revealed a vast range of E2-suppressed, antihormone-induced genes in addition to EGFR and ErbB2, which may also play a significant role in modulating response and resistance to endocrine therapies [2-5]. These genes include *ErbB3 *and *ErbB4*, the remaining members of the ErbB receptor family, which are receptors for the neuregulin (NRG) family of growth factors: NRG1 or heregulin (HRG), NRG2, NRG3 and NRG4 [2,22-24]. Like EGFR and ErbB2, ErbB4 can form active homodimers; however, ErbB3 is catalytically inactive and thus requires heterodimerization with another ErbB family member to promote signalling [25]. Both ErbB3 and ErbB4 have been shown to be E2-suppressed and tamoxifen-induced in ER-positive breast cancer cells, suggesting a potential role for both receptors in antihormone response and resistance [2,22]. This is supported by the findings of Tang and colleagues [26], who demonstrated that inoculation of HRG-transfected MCF-7 cells into the mammary fat pads of ovariectomised, athymic nude mice can generate oestrogen-independent and anti-oestrogen-resistant tumours. More recently, the weight of evidence has supported a role for ErbB3, rather than ErbB4, with increased activation of ErbB3 being reported in acquired tamoxifen- and fulvestrant-resistant MCF-7 cells [27,28] and its downregulation-enhancing responsiveness of ErbB2-overexpressing, *de novo *antihormone-resistant breast cancer cells to tamoxifen [29]. Moreover, ErbB1-ErbB3 overexpression has been reported to predict early relapse during tamoxifen therapy in ER-positive breast cancer patients [30,31]. The involvement of ErbB4 remains unclear, as in acquired tamoxifen-resistant MCF-7 cells expression of this receptor is enhanced [28], whereas in a panel of fulvestrant-resistant MCF-7 cells it is decreased [27]. Furthermore, in agreement with the fulvestrant resistance studies, loss of ErbB4 expression has been reported to be an independent marker of tamoxifen resistance in patients with primary breast cancer [32].

In the present study, we have examined the acute inductive capacity of the pure anti-ER fulvestrant on ErbB3 and ErbB4 receptor expression in a panel of four ER-positive breast cancer cell lines, two ErbB2-negative (MCF-7 and T47D) and two ErbB2-positive (BT474 and MDAMB361), and assessed the effect of ligand activation of these receptors on antihormone response. We demonstrate that seven-day fulvestrant treatment induces the expression of both the ErbB3 and ErbB4 receptors, resulting in enhanced sensitivity to the action of HRGβ1 and enabling this ligand to readily promote fulvestrant-resistant cell growth in all four cell lines.

## Materials and methods

### Cell culture

All tissue culture media and constituents were purchased from Gibco Europe Ltd. (Paisley, UK), and tissue culture plastics were obtained from Nunc (Roskilde, Denmark). A panel of four ER-positive breast cancer cell lines were used in this study: MCF-7, T47D, BT474 and MDAMB361. All of these cell lines were maintained in phenol red-free RPMI (wRPMI) medium containing 5% foetal calf serum (FCS), penicillin-streptomycin (10 IU/ml to 10 μg/ml), Fungizone (2.5 μg/ml), glutamine (4 mM) at 37°C in a humidified 5% CO_2 _atmosphere.

### Western blot analysis and reverse transcriptase polymerase chain reaction assay

#### Experimental cell culture

The four cell lines were grown in wRPMI supplemented with 5% FCS for seven days in the presence of fulvestrant (100 nM) alone, HRGβ1 (10 ng/ml) alone, a combination of the two agents or the appropriate vehicle control. Further studies were also performed where cell lines were grown in wRPMI supplemented with 5% FCS for seven days in either the absence or the presence of fulvestrant (100 nM) and subsequently exposed to either HRGβ1 (0.1 to 10 ng/ml in ethanol; Sigma, Poole, UK) or vehicle control for five minutes. All experiments were performed at least three times.

#### Protein cell lysis

Cells were washed three times with phosphate-buffered saline (PBS) and lysed using ice-cold lysis buffer (for composition used, see [12]). The cellular contents were transferred to microfuge tubes and clarified by centrifugation at 13,000 rpm for 15 minutes at 4°C, and supernatant aliquots were stored at -20°C until required. Total protein concentrations were determined using the DC Bio-Rad protein assay kit (Bio-Rad Laboratories Ltd., Hemel Hempstead, UK).

#### Western blot analysis

Protein samples from total cell lysates (50 μg) were subjected to electrophoretic separation on a 7.5% polyacrylamide gel and transblotted onto a nitrocellulose membrane (Schleicher and Schuell, Dassel, Germany). Blots were blocked at room temperature for one hour in 5% skimmed milk powder made up in Tris-buffered saline (TBS)-Tween 20 (TBS-T) (0.05%) and incubated for a minimum of one hour in primary antibody diluted 1:40,000 for β-actin (reference control) or 1:1,000 for epidermal growth factor receptor (EGFR), ErbB2, ErbB3, ErbB4, AKT, extracellular signal-regulated kinases 1 and 2 (ERK1/2), MAPK, protein kinase C and cyclin D1 in 1% MARVEL/TBS-T. The membranes were washed three times in TBS-T and then incubated for one hour with the required secondary immunoglobulin G horseradish peroxidase-labelled donkey anti-rabbit or sheep anti-mouse antibody (Amersham Biosciences UK Ltd., Buckinghamshire, UK), diluted 1:20,000 in 1% MARVEL/TBS-T. Detection was performed using West Dura chemiluminescence detection reagents (Pierce and Warriner Ltd., Chester, UK). Antibodies used were total EGFR (SC-03), ErbB2 (SC-284), ErbB3 (SC-285), ErbB4 (SC-283), phospho-ErbB4 (pY1056, SC-33040) and cyclin D1 (SC20044; Insight Biotechnology Ltd., Wembley, UK), as well as phospho-ErbB3 (pY1289, 4791), phospho-ErbB4 (pY1248, 4757), phospho-ErbB2 (pY1248, 2247), phospho-EGFR (pY1068, 2234), total AKT (9272), phospho-AKT (pS473, 9271), total ERK1/2 (9102) and phospho-ERK1/2 (pT202/pY204, 9101) (New England Biolabs, Hertfordshire, UK), ERα (ID-5) (Dako, Ely, UK) and β-actin (AC-15) (Sigma). These antibodies were selected as they have been demonstrated to be monospecific and do not cross-react with other family members.

#### Reverse transcriptase polymerase chain reaction

Total RNA was isolated from the four cell lines using an RNA isolator kit (TRI Reagent; Sigma), and 1 μg was reverse-transcribed using standard conditions as described previously [12]. The resultant cDNA samples were amplified using specific primers for progesterone receptor (PgR) and β-actin (housekeeping positive control), respectively, and conditions were optimised as described previously [12]. Briefly, an initial denaturing step of 95°C for two minutes was followed by a set number of cycles of 94°C for 30 seconds, 55°C for 30 seconds and 72°C for 30 seconds. PCR products were separated on a 3% wt/vol agarose gel containing ethidium bromide and visualised by ultraviolet illumination. The primers used for ErbB3, ErbB4, PgR and β-actin were reported previously [12].

### Cell proliferation

Cell monolayers were grown for 7 days in wRPMI supplemented with 5% FCS in the presence of either fulvestrant (100 nM), fulvestrant and gefitinib (1 µM; a kind gift from AstraZeneca, Macclesfield), fulvestrant and trastuzumab (100 nM; a kind gift from Roche Diagnostics, Penzburg), HRGβ1 (10 ng/ml), a combination of these agents or the appropriate vehicle control. Cell population growth was then evaluated by means of trypsin dispersion of the cell monolayers and cell number was measured using a Coulter counter (Luton, UK). All experiments were performed in triplicate.

### Immunocytochemistry

#### Experimental cell culture

The 4 cell lines were grown on sterile 3-aminopropyltriethoxysilane-coated coverslips at 1 × 10^4 ^cells/cm^2 ^in wRPMI supplemented with 5% FCS for 7 days in the presence of either fulvestrant (100 nM) alone, HRGβ1 (10 ng/ml) alone, a combination of the two agents or the appropriate vehicle control. The coverslips were then washed with PBS and fixed immediately according to the immunocytochemical assay to be performed. The Ki-67 assay used in these studies was performed according to the protocol previously described [33].

#### Assessment

Immunostaining for each assay was assessed by two personnel using a dual viewing attachment to an Olympus BH-2 light microscope (Southend-on Sea, Essex, UK). The percentage of cells that stained positive for nuclear Ki-67 was determined using a minimum evaluation of 2,000 cells/coverslip.

### Statistics

For immunocytochemical analysis, comparisons of the percentage of cells that stained positive for nuclear Ki-67 were determined using the Mann-Whitney *U *test for nonparametric data. For growth studies, overall differences between control and treatment groups in all four cell lines were determined by one-way analysis of variance. Direct comparisons between control and treatment effects were assessed using a *post hoc t*-test with either the Tamhane test or the Bonferroni adjustment factor for unequal and equal variances, respectively. Differences were considered significant at the *P *≤ 0.05 level.

## Results

### Anti-ER activity of fulvestrant in four ER-positive breast cancer cell lines

**Figure 1:**
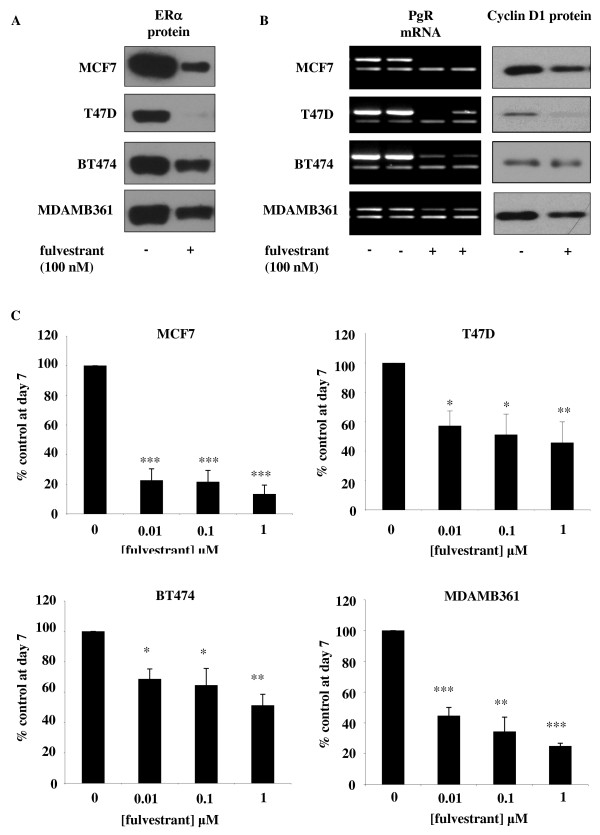
**Effect of fulvestrant on ER signalling and growth in MCF-7, T47D, BT474 and MDAMB361 cells**. Western blotting and reverse transcriptase-polymerase chain reaction (RT-PCR) analysis demonstrating effect of a seven-day incubation of MCF-7, T47D, BT474 and MDAMB361 cells with either fulvestrant (100 nM) or vehicle control (ethanol) on **(A) **oestrogen receptor α (ERα) protein and **(B) **progesterone receptor (PgR) mRNA, β-actin mRNAand cyclin D1 protein expression. For each cell line, PgR mRNA expression is shown as upper band and β-actin mRNA as lower band on RT-PCR image. Data are representative of at least three separate experiments. β-actin protein expression was also assessed for all Western blotting studies (data not shown) to confirm equivalent sample loading. **(C) **Effects of increasing concentrations of fulvestrant (0.01 to 1 μM) on the basal growth of MCF-7, T47D, BT474 and MDAMB361 cells on day 7 after initial treatment. The results are expressed as means ± SEM of triplicate wells and are representative of three separate experiments. *P < 0.05 versus control (0), **P < 0.01 versus control (0), ***P < 0.001 versus control (0).

Western blot analysis revealed that treatment of MCF-7, T47D, BT474 and MDAMB361 cell lines with fulvestrant at a concentration of 100 nM for seven days resulted in a substantial reduction in ERα protein levels in all four cell lines. The reduction was most apparent in MCF-7 and T47D cells, with expression of ER being virtually abolished; however, ERα expression was still clearly apparent, although greatly reduced, in fulvestrant-treated BT474 and MDAMB361 cells (Figure 1A). Loss of ERα expression in response to fulvestrant treatment was associated with reductions in PgR mRNA and cyclin D1 protein expression in all four cell lines, indicative of a reduction in ERα transcriptional activity (Figure 1B). Cell proliferation was also significantly reduced by fulvestrant treatment in a concentration-dependent manner in all four cell lines with this pure anti-ER, with the maximal concentration being 100 nM, significantly reducing growth by about 90% in MCF-7 cells and approximately 80% in MDAMB361 cells (*P *< 0.001, *n *= 3 for both cell lines) (Figure 1C) and by about 60% in T47D cells and approximately 50% in BT474 cells (*P *< 0.01, *n *= 3 for both cell lines) (Figure 1C). These decreases in cell numbers were mirrored by similar reductions in nuclear Ki-67 immunostaining across the four cell lines, with 100 nM fulvestrant significantly lowering nuclear Ki-67 positivity, expressed as mean ± SD, from 97 ± 1.2% to 55 ± 4.5% in MCF-7 cells (*P *< 0.01, *n *= 3), 80 ± 2.2% to 41 ± 3.6% in T47D cells (*P *< 0.001, *n *= 3), 38 ± 2.2% to 13 ± 1.3% in BT474 cells (*P *< 0.001, *n *= 3) and 82 ± 0.9% to 38 ± 1.8% in MDAMB361 cells (*P *< 0.001, *n *= 3) (Table 1 and Additional file 1, Supplementary Figure S1).

**Table 1:** Effects of fulvestrant, HRGβ1 or a combination of the two treatments on immunocytochemically determined nuclear Ki-67 positivity in MCF-7, T47D, BT474 and MDAMB361 cells

	Nuclear Ki-67 positivity, %			
	
Cell type	Control	Fulvestrant	HRGβ1	Fulvestrant + HRGβ1
MCF-7	97 ± 1.2	55 ± 4.5*	90 ± 0.4	89 ± 1.8†
T47D	80 ± 2.2	41 ± 3.6*	63 ± 2.7**	49 ± 5.8
BT474	38 ± 2.2	13 ± 1.3*	48 ± 2.2***	50 ± 1.3††
MDAMB361	82 ± 0.9	38 ± 1.8*	59 ± 2.2**	71 ± 2.2††

HRGβ1, heregulin β1. Nuclear positivity percentage values are expressed as means ± standard deviation of the assessment of six fields of view per coverslip in triplicate experiments.

*P < 0.001 versus control, **P < 0.05 versus control, ***P < 0.01 versus control, ^†^*P *< 0.01 versus fulvestrant, ^††^*P *< 0.001 versus fulvestrant.

#### Induction of ErbB3 and ErbB4 receptor expression and signalling by fulvestrant

**Figure 2:**
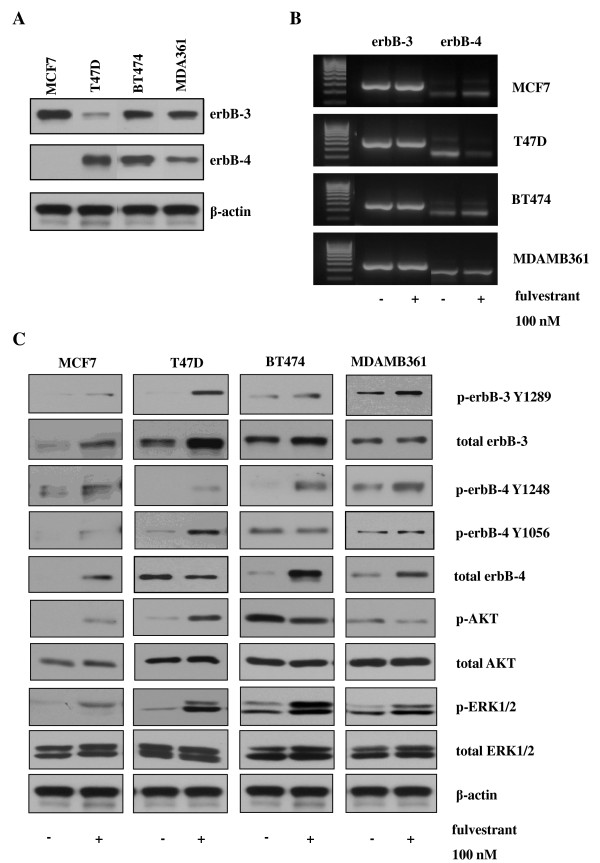
**Effect of fulvestrant on erbB3/4 expression and activity in MCF-7, T47D, BT474 and MDAMB361 cells**. **(A) **Western blots showing erbB3 and erbB4 protein expression in MCF-7, T47D, BT474 and MDAMB361 cells maintained in phenol red-free RPMI medium containing 5% foetal calf serum. **(B) **Reverse transcriptase polymerase chain reaction (RT-PCR) analysis of erbB3 and erbB4 mRNA expression. **(C) **Western blot analysis of total and phosphorylated erbB3, erbB4, AKT and extracellular signal-regulated kinases 1 and 2 (ERK1/2) protein expression in MCF-7, T47D, BT474 and MDAMB361 cells prior to and following treatment with fulvestrant (100 nM) for seven days. β-actin was used as a loading control (not shown for RT-PCR).

Expression of ErbB3 protein was observed across the panel of cell lines; however, ErbB4 protein was detected only in the T47D, BT474 and MDAMB361 cell lines (Figure 2A). The expression profile for both ErbB receptors was highly heterogeneous across the four cell lines. In the ErbB2 low-expressing MCF-7 and T47D cell lines, seven-day fulvestrant treatment at a concentration of 100 nM promoted ErbB3 protein but not mRNA expression in both cell lines (Figures 2B and 2C). ErbB4 mRNA and protein expression increased in response to fulvestrant in MCF-7 cells; however, in T47D cells, ErbB4 mRNA and protein expression were clearly reduced in response to treatment with this pure anti-ER (Figures 2B and 2C). In the ErbB2-positive BT474 and MDAMB361 cell lines, ErbB3 mRNA and protein expression appeared unchanged following treatment with fulvestrant, whilst ErbB4 protein but not mRNA expression was clearly enhanced in response to this antihormone (Figures 2B and 2C).

In addition to promoting ErbB3 and ErbB4 receptor protein expression, fulvestrant treatment also enhanced basal phosphorylation of both ErbB receptor family members in all four cell lines (Figure 2C). Two ErbB4 tyrosine (Y) phosphorylation sites, Y1284 and Y1056, were examined, with increased levels of Y1284 phosphorylation seen across all four cell lines following fulvestrant treatment and increased Y1056 phosphorylation being observed in the ErbB2 low-expressing but not in the ErbB2-overexpressing cell lines in response to the antihormone (Figure 2C). This enhanced basal ErbB3 and ErbB4 activity was also associated with increased basal phosphorylation of ERK1/2 in the four cell lines, with the antihormone having no effect on total ERK1/2 expression (Figure 2C). A differential effect of fulvestrant on AKT activity was observed in the ErbB2 low-expressing compared to ErbB2-overexpressing cell lines, with fulvestrant promoting basal AKT phosphorylation in MCF-7 and T47D cells but causing, if anything, a small reduction in phosphorylation of this signalling element in the BT474 and MDAMB361 cell lines (Figure 2C).

### Fulvestrant sensitises ER-positive cell lines to HRβ1

#### Effect of HRGβ1 in the absence of fulvestrant

**Figure 3:**
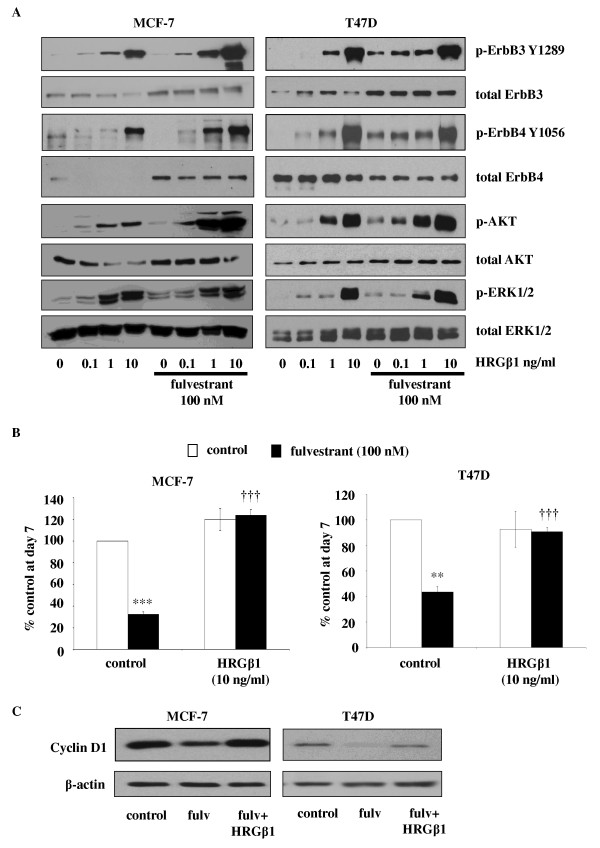
**Effect of fulvestrant on heregulin β1 (HRGβ1) signalling in MCF-7 and T47D cells**. **(A) **Western blots showing the effect of increasing concentrations of heregulin β1 (HRGβ1) (0.1 to 10 ng/ml for five minutes) on total and phosphorylated ErbB3, ErbB4, AKT and extracellular signal-regulated kinases 1 and 2 (ERK1/2) protein expression in MCF-7 and T47D cells, maintained for 7 days in the presence of either fulvestrant (100 nM) of vehicle control (ethanol). **(B) **Graphs showing cyclin D1 protein expression in MCF-7 and T47D cells maintained for seven days in the presence of either fulvestrant or vehicle control and subsequently exposed to either HRGβ1 or vehicle control for five minutes. β-actin was used as a loading control. **(C) **Western blots showing the effect of HRGβ1 (10 ng/ml) on growth of MCF-7 and T47D cells maintained for seven days in the presence of either fulvestrant or vehicle control (ethanol). The results are expressed as means ± SEM of triplicate wells and are representative of three separate experiments. **P < 0.01 versus control, ***P < 0.001 versus control, †††*P *< 0.001 versus fulvestrant.

In the MCF-7 and T47D cell lines, HRGβ1 induced a concentration-dependent activation of ErbB receptors and associated downstream signalling elements, with phosphorylation of both ErbB3, ErbB4, ERK1/2 and AKT clearly apparent following exposure to 1 to 10 ng/ml HRGβ1 (Figure 3A). Interestingly, HRGβ1 phosphorylated ErbB4 predominantly at Y1056 in these cells, with little effect on Y1284 being observed (data not shown). Activation of these signalling pathways by HRGβ1 was associated with a trend towards increased proliferation of MCF-7 but not T47D cells (Figure 3B). Thus, 10 ng/ml HRGβ1 caused a 20% to 30% increase in cell numbers; however, no obvious increase in Ki-67 immunostaining could be observed, as nuclear expression of this protein in control MCF-7 cells was close to 100% (Figure 3B, Table 1 and Additional file 1, Supplementary Figure S1). No significant change in T47D cell numbers was observed following HRGβ1 treatment; however, a small but significant reduction in nuclear Ki-67 staining from 80% to 63% was noted in response to this ligand (*P *< 0.05, *n *= 3) (Figure 3B, Table 1 and Additional file 1, Supplementary Figure S1).

**Figure 4:**
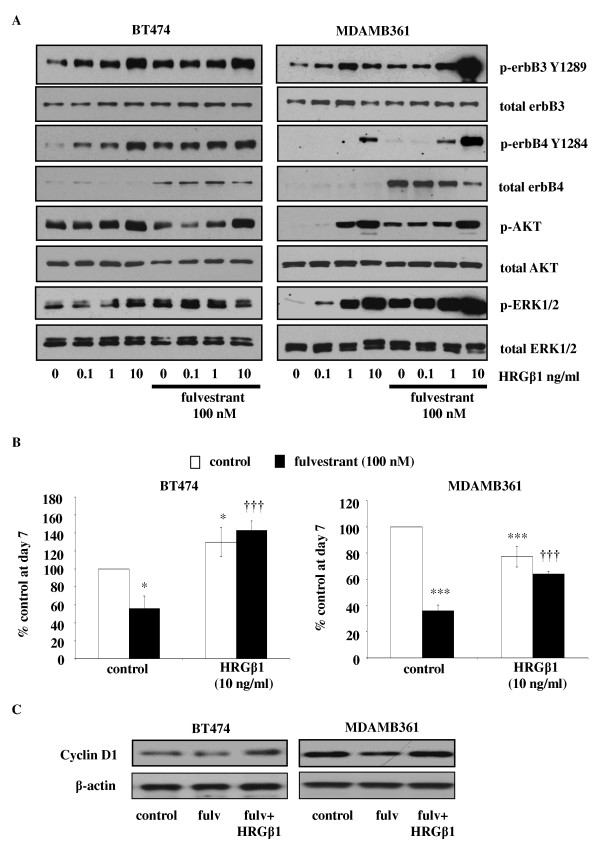
**Effect of fulvestrant on heregulin β1 (HRGβ1) signalling in BT474 and MDAMB361 cells**. **(A) **Western blots showing the effect of increasing concentrations of heregulin β1 (HRGβ1) (0.1 to 10 ng/ml for five minutes) on total and phosphorylated ErbB3, ErbB4, AKT and extracellular signal-regulated kinases 1 and 2 (ERK1/2) protein expression in BT474 and MDAMB361 cells, maintained for 7 days in the presence of either fulvestrant (100 nM) of vehicle control (ethanol). **(B) **Graphs showing cyclin D1 protein expression in BT474 and MDAMB361 cells maintained for seven days in the presence of either fulvestrant or vehicle control and subsequently exposed to either HRGβ1 or vehicle control for five minutes. β-actin was used as a loading control. **(C) **Western blots showing the effect of HRGβ1 (10 ng/ml) on the growth of BT474 and MDAMB361 cells maintained for seven days in the presence of either fulvestrant (100 nM) or vehicle control (ethanol). The results are expressed as means ± SEM of triplicate wells and are representative of three separate experiments. *P < 0.05 versus control, ***P < 0.001 versus control, †††*P *< 0.001 versus fulvestrant.

In the MDAMB361 and BT474 cell lines, HRGβ1 similarly promoted a concentration-dependent activation of ErbB3, ErbB4, AKT and ERK1/2 (Figure 4A); however, in these cell lines, HRGβ1 phosphorylated ErbB4 primarily on Y1284 (Figure 4A), with little phosphorylated Y1056 observed (data not shown). BT474 cells appeared less responsive than MDAMB361 cells to HRGβ1 with respect to activation of AKT and ERK1/2, with increased activity of both these elements being seen only in response to the highest concentration of this ligand (10 ng/ml). Interestingly, activation of these signalling pathways had diametrically opposite effects on proliferation of the two cell lines. Thus, HRGβ1 was a modest growth suppressor of MDAMB361 cells, with a 10 ng/ml concentration significantly decreasing cell number by about 20% (*P *< 0.01, *n *= 3) and significantly reducing Ki-67 staining, expressed as mean ± SD, from 82 ± 0.9% to 59 ± 2.2% (*P *< 0.001, *n *= 3), but a growth promoter of BT474 cells with cell number significantly increasing by approximately 20% (*P *< 0.05, *n *= 3) and nuclear Ki-67 positivity rising significantly from 38 ± 2.2% to 48 ± 2.2% in response to 10 ng/ml HRGβ1 (P < 0.001, *n *= 3) (Figure 4B, Table 1 and Additional file 1, Supplementary Figure S1).

#### Effects of HRGβ1 in the presence of fulvestrant

**Figure 5:**
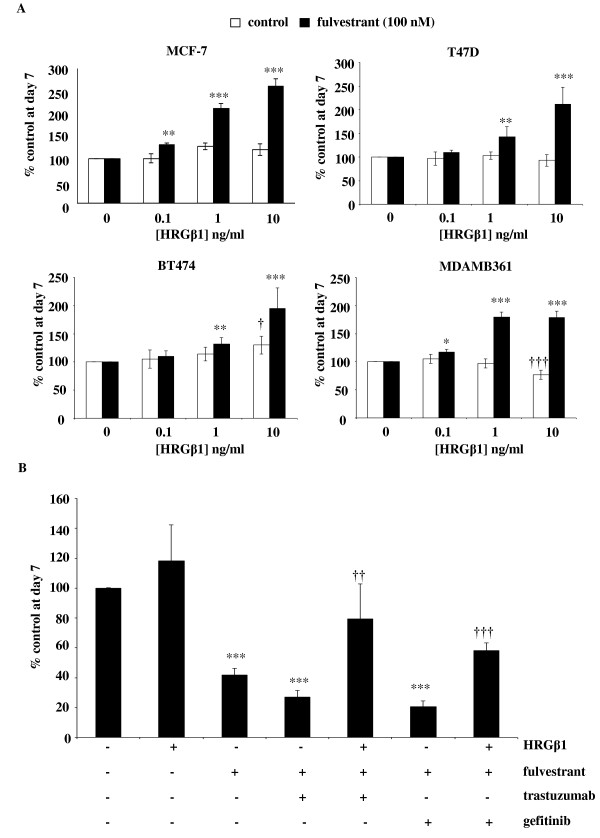
**Effect of heregulin β1 (HRGβ1) on growth of fulvestrant-treated MCF-7, T47D, BT474 and MDAMB361 cells**. **(A) **Effect of heregulin β1 (HRGβ1) (0.1-10 ng/ml) on growth of MCF-7, T47D, BT474 and MDAMB361 cells maintained for seven days in the presence of either fulvestrant (100 nM) or vehicle control (ethanol). *P < 0.05 versus control (0), **P < 0.01 versus control (0), ***P < 0.001 versus control (0). **(B) **Effect of HRGβ1 (10 ng/ml) on growth of MCF-7, T47D, BT474 and MDAMB361 cells maintained for seven days in the presence of fulvestrant alone (100 nM), fulvestrant in combination wither either gefitinib (1 µM) or trastuzumab (100 nM) or appropriate vehicle control. The results are expressed as means ± SEM of triplicate wells and are representative of three separate experiments. ***P < 0.001 versus no treatment, ††*P *< 0.01 versus fulvestrant + herceptin, †††*P *< 0.001 versus fulvestrant + gefitinib.

Following a seven-day treatment with fulvestrant, the MCF-7 and T47D cell lines demonstrated enhanced sensitivity to increasing concentrations of HRGβ1, with phosphorylation of ErbB receptors ERK1/2 and AKT observed at the lower concentration of 0.1 ng/ml HRGβ1 and a greater magnitude of phosphorylation apparent in response to the higher concentrations of the ligand (Figures 3A). This enhanced signalling response to HRGβ1 was associated with recovery of cyclin D1 protein expression and enhanced proliferative activity with HRGβ1 treatment completely overriding the growth-inhibitory effects of fulvestrant in both cell lines (*P *< 0.001, *n *= 3) (Figures 3B and 3C). Indeed, fulvestrant treatment converted HRGβ1 from a ligand with limited or negligible proliferative activity to one that potently and significantly promoted cell growth in both cell lines (*P *< 0.001, *n *= 3) (Figure 5A). This was clearly reflected in the Ki-67 immunostaining of MCF-7 cells, with HRGβ1 increasing nuclear Ki-67 positivity scores, expressed as mean ± SD, from 55 ± 4.5% to 89 ± 1.8% in MCF-7 cells (*P *< 0.01, *n *= 3); however, in T47D cells, although an increase from 41 ± 3.6% to 49 ± 5.8% was observed in response to HRGβ1 in the presence of fulvestrant, it did not reach statistical significance (Table 1 and Additional file 1, Supplementary Figure S1).

Enhanced sensitivity and an increased magnitude of response to HRGβ1 was also observed in MDAMB361 and BT474 cells following fulvestrant exposure, with enhanced phosphorylation levels of ErbB4 and ERK1/2 being observed in both cell lines in response to increasing concentrations of this ligand (Figure 4A). HRGβ1-induced activation of ERK1/2 was less apparent in BT474 cells compared to MDAMB361 cells, but a small increase in ERK1/2 activity in response to the lower concentrations of the ligand, particularly 0.1 ng/ml, was still apparent in the BT474 cell line, indicative of a sensitisation effect of fulvestrant in these cells. Enhanced HRGβ1-induced ErbB3 phosphorylation was also seen in the MDAMB361 cells but not the BT474 cells in the presence of fulvestrant (Figure 4A). Interestingly, in both cell lines, there was no sensitisation of AKT activity in response to HRGβ1; if anything, there appeared to be a reduced response to the lower concentrations of this ligand (0.1 to 1 ng/ml) following fulvestrant treatment, which was most apparent in the BT474 cell line (Figure 4A). As a consequence of the enhanced HRGβ1-induced signalling seen in both cell lines in the presence of fulvestrant, once again, this ligand was able to promote recovery of cyclin D1 protein expression and potently and significantly overcome the growth-inhibitory effects of the antihormone in both cell lines (*P *< 0.001, *n *= 3) (Figures 4B and 4C). Furthermore, as observed in the ErbB2 low-expressing cell lines, fulvestrant treatment significantly enhanced the proliferative activity of HRGβ1 in BT474 cells and converted this ligand from one that suppressed growth into one that potently and significantly promoted MDAMB361 cell growth (*P *< 0.001, *n *= 3) (Figure 5A). This ability of HRGβ1 to promote growth in both cell lines in the presence of fulvestrant was again reflected in Ki-67 immunostaining, with nuclear Ki-67 positivity, expressed as mean ± SD, significantly rising from 13 ± 1.3% to 50 ± 1.3% in BT474 cells and from 38 ± 1.8% to 71 ± 2.2% in MDAMB361 cells in the presence of a 10 ng/ml concentration of this ligand (*P *< 0.001, *n *= 3 for both cell lines) (Table 1 and Additional file 1, Supplementary Figure S1).

#### Effects of HRGβ1 in the presence of fulvestrant in combination with either gefitinib or herceptin

Treatment of MCF-7 cells with a combination of fulvestrant and either gefitinib (1 µM) or herceptin (100 nM) for seven days significantly and potently reduced cell growth (*P *< 0.001, *n *= 3). Although this result was not statistically significant, there was a trend towards the combination's providing a greater inhibition of cell growth compared to fulvestrant alone. However, this enhanced growth-inhibitory action of the combination treatments did not achieve statistical significance compared with the fulvestrant-alone arm (Figure 5B). Importantly, HRGβ1 treatment was capable of a partial but statistically significant increase in cell growth in the presence of both fulvestrant and herceptin (*P *< 0.01, *n *= 3) and fulvestrant and gefitinib (*P *< 0.01, *n *= 3) combination therapies in this cell line (Figure 5B).

## Discussion

Antihormonal therapy has proved to be highly successful in the treatment of ER-positive breast cancer; however, resistance to these agents remains a significant clinical problem, with many patients either gaining no benefit or relapsing during therapy [15]. Numerous preclinical and clinical studies have established that increased expression of two members of the ErbB receptor family, EGFR and ErbB2, plays a central role in the acquisition of resistance to antihormonal therapies [12-19]. Upregulation of EGFR and ErbB2 has been reported to be an early response to antihormone treatment in ER-positive breast cancer cell lines [2-4,6-11]; however, despite a number of preclinical findings reporting improved magnitude and duration of response with combined targeting of EGFR/ErbB2 and ER signalling [21], translation of these findings into the clinical setting has proved largely disappointing, with a large number of patients gaining little or no benefit from such combination therapies [20]. Further studies have now revealed an array of candidate genes with potential involvement in antihormone resistance, including the other members of the ErbB receptor family, ErbB3 and ErbB4. As both ErbB3 and ErbB4 have been identified as oestrogen-suppressed and antihormone-induced genes in MCF-7 breast cancer cells [22], we have examined whether upregulation of these receptors is an early response to the pure anti-ER fulvestrant in a panel of four ER-positive breast cancer cell lines, two HER2-overexpressing and two HER2 low-expressing, and if so, what effect ligand activation of these receptors has on the acute growth-inhibitory activity of this antihormone.

Fulvestrant consistently inhibited growth of all four ER-positive cell lines at day 7 post-antihormone treatment, with MCF-7 and MDAMB361 cells demonstrating a greater sensitivity than T47D and BT474 cells to this pure anti-ER. In all four cell lines, the blockade of growth was associated with substantial reductions in ER protein expression with resultant decreased transcriptional activity, as evidenced by decreased PgR mRNA and cyclin D1 protein expression levels. These findings clearly indicate the potent anti-ER activity of fulvestrant and are consistent with previous reports of fulvestrant action in ER-positive breast cancer cell lines [34-36]. However, alongside this potent acute growth-inhibitory activity of fulvestrant, there was also clear evidence of the inductive capacity of this agent, with increased expression of ErbB3 protein expression in the MCF-7 and T47D cells and ErbB4 protein expression in MCF-7, BT474 and MDAMB361 cell lines at day 7 post-antihormone treatment. Although ErbB3 and ErbB4 have previously been reported to be ER-suppressed, antihormone-induced genes in MCF-7 cells [22], we believe that the present article is the first to report acute induction of these receptors at the protein level by antihormonal therapy in a panel of HER2 low-expressing and overexpressing ER-positive breast cancer cell lines. Interestingly, there was no consistent upregulation of either the ErbB3 or ErbB4 receptor across the panel of cell lines; however, increased ErbB3 expression was common to the ErbB2 low-expressing cells, whilst enhanced ErbB4 levels were common to the two ErbB2-overexpressing cell lines. In the T47D cell line, a reduction in ErbB4 mRNA and protein expression was observed following fulvestrant treatment, consistent with previous reports that ErbB4 is an ER-induced and, by implication, antihormone-suppressed gene in this cell line [23]. It is also important to note that the increase in ErbB3 protein expression in MCF-7 and T47D cells and the increase in ErbB4 protein expression in BT474 and MDAMB361 cells in response to fulvestrant were not mirrored by any substantial changes in mRNA expression of these receptors, findings that are fully supported by Affymetrix gene array analysis of the expression of these ErbB receptors in the four cell lines prior to and following 10-day fulvestrant treatment (data not shown). This would suggest that this acute inductive response to fulvestrant is mediated predominantly by a posttranscriptional, posttranslational mechanism.

Although research into the role of ErbB receptor signalling in breast cancer has focused primarily on EGFR and ErbB2, it is becoming increasingly clear that both ErbB3 and ErbB4 also have important roles to play in this disease. ErbB3 has been identified as a key partner for ErbB2, with this receptor heterodimer being identified as an oncogenic unit in breast cancer cells [37], whilst overexpression of ErbB4 has also been shown to promote growth of human breast cancer cells [38,39]. Furthermore, blockade of ErbB3 expression using an artificial transcription factor, E3, inhibits breast cancer cell growth and targeted downregulation of ErbB4, using either ribozymes or small interfering RNA, can reduce the growth of MCF-7 and T47D cell lines both *in vitro *and *in vivo *[23,40]. Both receptors have also been shown to be overexpressed in breast cancer; however, their prognostic significance remains a subject of debate. High expression of ErbB3 has been shown to have a positive association with tumour size, recurrence, metastasis and significantly reduced patient survival [41-46]. However, ErbB3 receptor status has also been reported to have a positive prognostic value by associating with an ER-positive phenotype and longer disease-free survival for breast cancer patients [45,47,48]. Similarly, an association between the expression of ErbB4 and a favourable clinical outcome has been reported in some clinical studies [41,45,48,49], whilst other researchers have suggested that ErbB4 expression might be a marker of a poorer outcome in some breast cancer patients [43,50]. These discrepancies are probably due to the fact that the function of these receptors is highly reliant on their localisation and the relative expression levels of other ErbB receptor family members within the tumour cells. For example, both ErbB3 and ErbB4 have been found to localise not only at the membrane but also within the nucleus in breast cancer cells [51,52]. The role of nuclear ErbB3 remains unclear; however, levels are higher in nonmalignant versus malignant breast epithelial cells, suggesting that it may have potential antiproliferative activity [51]. In contrast, ErbB3 localised at the membrane can heterodimerize with other ErbB family members, principally ErbB2, in response to ligand stimulation to potently promote breast cancer cell growth [37]. In the case of ErbB4, nuclear expression of the intracellular domain of this receptor generated by the proteolytic actions of tumour necrosis factor-α-converting enzyme and γ-secretase following ligand binding promotes ER-driven cell growth [23,52], whilst cytosolic and membrane localisation of ErbB4 has been shown to inhibit growth and promote apoptosis in breast cancer cells [53-55]. This varied function according to ErbB receptor expression and localisation may also go some way toward explaining the differential effect of HRGβ1 on the growth of the four breast cancer cell lines we observed in the present study. In MCF-7 and BT474 cells, HRGβ1 promoted cell growth, whilst this ligand had little effect on T47D cell proliferation and actually suppressed growth of MDAMB361 cells. A possible explanation for this is that the antiproliferative activity of ErbB4 may be more prominent in the T47D and MDAMB361 cell lines, as HRGβ1-induced activation of this receptor relative to ErbB3 was far greater in these cells compared to the other two cell lines.

In addition to enhancing total protein expression levels, fulvestrant treatment also promoted basal ErbB3 and ErbB4 phosphorylation in all four cell lines, which indicates enhanced signalling through these ErbB receptors. Indeed, the enhanced level of ErbB3 and ErbB4 phosphorylation in the MCF-7 and T47D cell lines was associated with increased activation of both the MAPK/ERK1/2 and PI3K/AKT signalling pathways, whilst in the BT474 and MDAMB361 cell lines, it was associated with increased ERK1/2 but not AKT activity, possibly reflecting the continued dominant role of ErbB2, a key recruiter of the MAPK pathway, in these cells. This induction of ErbB3 and ErbB4 signalling by fulvestrant, as previously suggested for EGFR and ErbB2, may provide these cells with a further input into signalling pathways that could potentially allow cells to survive the initial action of the antihormone and ultimately provide a resistance mechanism [5]. Indeed, this is supported by our finding that fulvestrant treatment enhanced the ability of HRGβ1 to stimulate signalling via ErbB3 and ErbB4, resulting in this ligand's potently overriding the suppression of cyclin D1 protein expression and the blockade of growth by fulvestrant in all four cell lines. Importantly, following fulvestrant treatment, HRGβ1 was converted from a ligand having quite differential effects on cell growth into one that consistently and potently promoted proliferation in all four cell lines examined. Interestingly, the mechanisms by which HRGβ1 overcame fulvestrant action were subtly different in the HER2 low-expressing and HER2-overexpressing cell lines. In the MCF-7 and T47D cells, fulvestrant selectively enhanced the ability of HRGβ1 to phosphorylate ErbB4 at Y1056, a PI3K-p85 recruitment site [56], and promote AKT signalling activity, whereas in the BT474 and MDAMB361 cells, it was the HRGβ1-induced ErbB4 Y1284 SHC recruitment site [56] and ERK1/2 phosphorylation that were selectively augmented by antihormonal treatment. The differential ErbB4 phosphorylation and downstream pathway recruitment may reflect the expression of alternative ErbB4 isoforms in these cells, with the CYT-2 isoform, which lacks the Y1056 PI3K binding consensus site, potentially being preferentially expressed in the ErbB2-overexpressing cell lines [57]. It is also possible that such differences may arise from distinct heterodimerization partners binding ErbB4 in response to HRGβ1 in the ErbB2 low-expressing and ErbB2-overexpressing cell lines following fulvestrant treatment. Researchers in ongoing studies are currently examining these possibilities. In MCF-7 cells, it seems that the likely heterodimerization partner for ErbB4 is ErbB3, as blockade of either EGFR with gefitinib or ErbB2 with herceptin was without effect on the ability of HRGβ1 to promote cell growth. Importantly, these findings also indicate that HRGβ1 signalling via ErbB3 and ErbB4 can provide a potent mechanism of resistance to such combination therapies and may provide an explanation for the disappointing results of clinical studies in which the combination strategy of antihormones alongside an anti-EGFR or anti-ErbB2 agent was examined [20,21]. These findings are identical to those of Sonne-Hansen and colleagues [58], who demonstrated that HRGβ1 can override the growth-inhibitory effects of fulvestrant in combination with cetuximab, trastuzumab or pertuzumab in MCF-7 cells. This group also showed that combining fulvestrant with a pan-ErbB inhibitor effectively prevented HRGβ1-induced growth in this cell line, further emphasising the key findings of the present study: that ErbB3 and ErbB4 have the potential to play a central role in the development of resistance to this antihormonal therapy [58]. However, it should also be noted that all of these studies were performed in a single cell line and that further studies in the other ER-positive cell lines are required to fully support this proposition.

## Conclusions

These current findings demonstrate that targeting ER signalling with the pure anti-ER fulvestrant can both suppress and induce gene and protein expression in ER-positive, ErbB2 low-expressing and ErbB2-overexpressing breast cancer cell lines. As expected, fulvestrant was a potent growth inhibitor in all four of these ER-positive breast cancer cell lines through its ability to suppress proliferation-related genes such as cyclin D1. However, the simultaneous induction of ErbB3 and ErbB4 also provided a mechanism for these cells, when in a HRGβ1-enriched environment, to promote reexpression of cyclin D1 and ultimately drive fulvestrant-resistant cell growth. What is more, fulvestrant treatment converted HRGβ1 from a ligand with both growth-promoting and growth-suppressive activity, depending on cell type, into one that consistently and potently promoted cell growth, regardless of ErbB2 status. Thus, although antihormones such as fulvestrant may have potent, acute growth-inhibitory activity in ER-positive breast cancer cells, their ability to rapidly induce and sensitize cells to growth factors such as HRGs may serve to reduce and ultimately limit their inhibitory activity. Indeed, such a rapid induction of these proliferative genes may provide a mechanism of *de novo *endocrine resistance in situations in which HRG expression in the tumour microenvironment is high.

## Abbreviations

E2: oestradiol; EGFR: epidermal growth factor receptor; ER: oestrogen receptor; ERK1/2: extracellular signal-regulated kinases 1 and 2; FCS: foetal calf serum; HRGβ1: heregulin β1; MAPK: mitogen-activated protein kinase; NRG: neuregulin; PBS: phosphate-buffered saline; PCR: polymerase chain reaction; PgR: progesterone receptor; PI3K: phosphatidylinositol 3-kinase; TBS: Tris-buffered saline; wRPMI: phenol red-free Roswell Park Memorial Institute; Y: tyrosine.

## Competing interests

IRH, JMWG and RIN are in receipt of funding from AstraZeneca. HEF is funded by a BBSRC AstraZeneca CASE studentship. RIN is also a member of an advisory board for AstraZeneca. The remaining authors declare that they have no competing interests.

## Authors' contributions

IRH conceived the study, participated in its design and execution and drafted the manuscript. LG and JMK carried out the Western blotting studies. HEF performed the RT-PCR analysis. RM and DB performed all cell cultures and carried out the growth studies. JMWG carried out the immunocytochemistry and with RIN participated in the design and coordination of the study and helped to draft the manuscript. All authors read and approved the manuscript.

## Supplementary Material

Additional file 1 **Supplementary Figure S1**. Effect of HRGβ1 (10 ng/ml) on nuclear Ki-67 immunostaining in MCF-7, T47D, BT474 and MDAMB361 cells maintained for 7 days in the presence of either fulvestrant (100 nM) or vehicle control (ethanol). Click here for file
