# Supplementary figures and images for: Fulvestrant-induced expression of ErbB3 and ErbB4 receptors sensitizes oestrogen receptor-positive breast cancer cells to heregulin β1
Source: Breast Cancer Res. 2011 Mar 11;13(2):R29. doi: 10.1186/bcr2848 (PMC3219190; doi:10.1186/bcr2848)

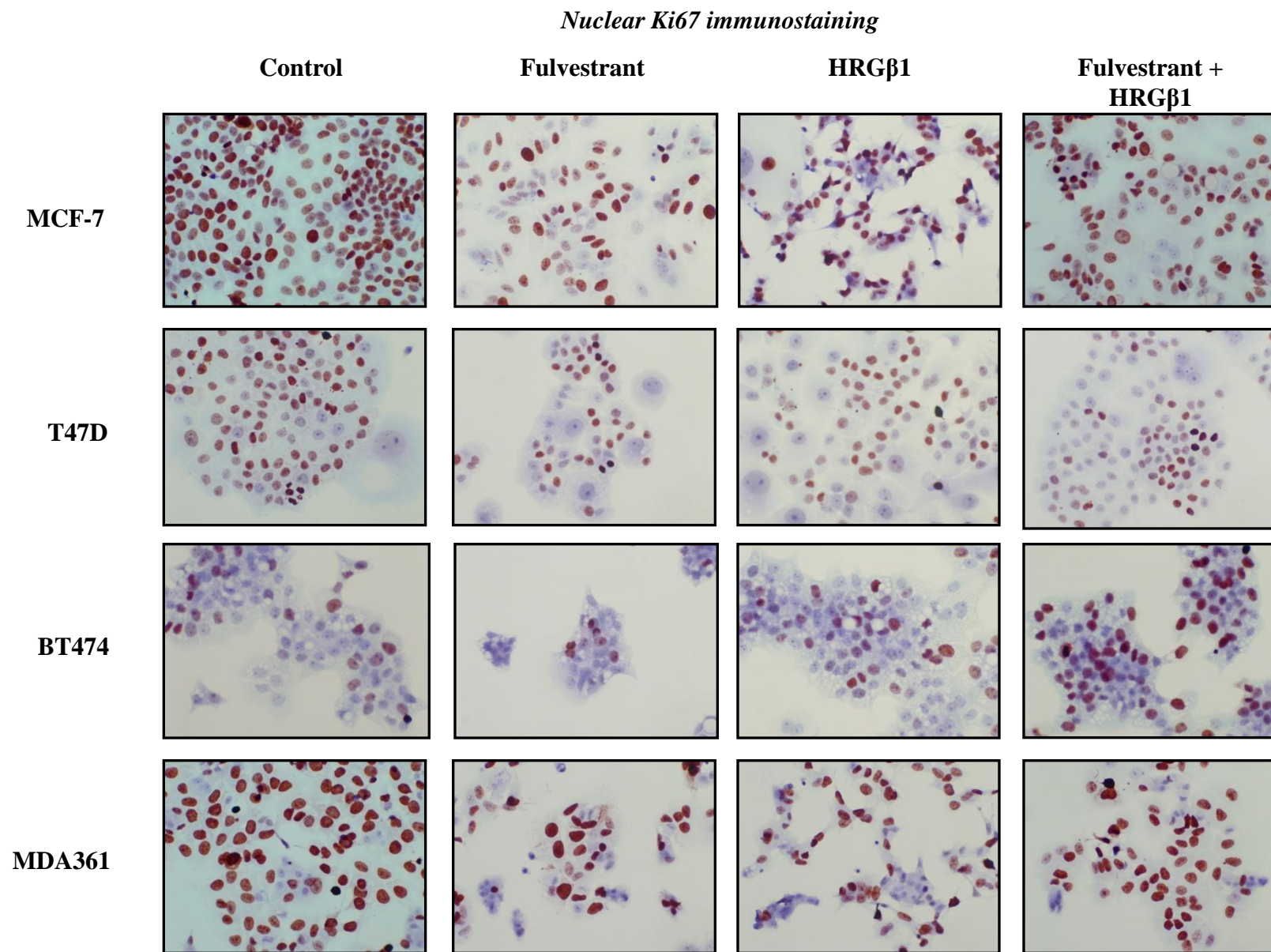

Supplement: Additional file 1 — Supplementary Figure S1. Effect of HRGβ1 (10 ng/ml) on nuclear Ki-67 immunostaining in MCF-7, T47D, BT474 and MDAMB361 cells maintained for 7 days in the presence of either fulvestrant (100 nM) or vehicle control (ethanol). [file bcr2848-S1.PDF]
